# Health of Youth in Transition in Hong Kong

**DOI:** 10.3390/ijerph17113791

**Published:** 2020-05-27

**Authors:** Ka-Man Leung, Folake Orekoya, Adrian J. Bailey, Hor-Yan Lai, Ka-Yi Chan, Ting-Lok Lam

**Affiliations:** 1 Department of Health and Physical Education, University of Hong Kong, Hong Kong, China; 2 Department of Sport and Physical Education, Hong Kong Baptist University, Hong Kong, China; rockhaven50@gmail.com; 3 Faculty of Social Sciences, Hong Kong Baptist University, Hong Kong, China; bailey@hkbu.edu.hk; 4 Department of Social Work, Hong Kong Baptist University, Hong Kong, China; angelhylai@hkbu.edu.hk; 5 Centre for the Advancement of Social Sciences, Hong Kong Baptist University, Hong Kong, China; channey@hkbu.edu.hk (K.-Y.C.); william_lam@hkbu.edu.hk (T.-L.L.)

**Keywords:** physical activity, adolescents, social ecological model

## Abstract

This study aimed to examine the impact of individual (level of vigorous physical activity (VPA) and frequency of using sports and recreation facilities), interpersonal (perceived social cohesion (PSC)), and neighborhood environmental (availability of sports and recreation facilities) factors on youths’ health in transition in Hong Kong. A sample of 508 individuals aged 17–23 years from all Hong Kong council districts randomly completed validated questionnaires by telephone survey. Of 508,302 individuals with complete data pertaining to address geocoding were selected for further analyses. Overall, more than half of them (56.3%) used sports and recreation facilities once per month or less. Structural equation modeling was used to examine the relationship among the studies’ constructs. The results indicated that the proposed model sufficiently fitted the data (χ^2^ (24) = 32.23, *p* < 0.12; CFI = 0.977; SRMR = 0.051; RMSEA = 0.034 (90% CI = 0.000 to 0.061). However, two items of PSC were sequentially removed due to their low standardized factor loadings (<0.3). A structural model was reinserted into data analyses, and the modified model fitted the data well as indicated by fit indices (χ^2^ (11) = 15.29, *p* < 0.17; CFI = 0.987; SRMR = 0.054; RMSEA = 0.036 (90% CI = 0.000 to 0.075). Only VPA (*β* = 0.27, *p* = 0.0005) and PSC (*β* = 0.12, *p* = 0.048) were significantly related to perceived health at an individual level. To promote youth health, the Hong Kong government may work with the business sector, community groups, or education institutions to develop community programs to keep youths active (especially VPA) and to build more cohesive, trustful relationships among youths in the neighborhood.

## 1. Introduction

The key stages that individuals go through in their lives have a particular relevance on health levels. The life course approach acknowledges the importance of the effect of physical and social exposures as well as physical and psychological changes in the transition from adolescence to adulthood [1]. These changes include increasing emotional and behavioral autonomy [2] and taking responsibility for his or her own decisions, including exercise habits and types and quantity of meals to consume [3]. In addition, during this transition students may transit from secondary school to university; as a part of this process they leave home, make new friends, and develop a new lifestyle [2]. Furthermore, transitioning from adolescence to adulthood involves individuals themselves, their families, as well as pertinent health community services [4]. This life course approach has been used to study chronic disease epidemiology [5] and health [1].

Youth health has suffered some setbacks in Hong Kong with a reduction in extracurricular activities, from 32.7% in 2001 to 25.7% in 2011 among 15–29-year-old individuals [6]. Herein, the term “youth” is defined as the period from early adolescence (10 years of age) to young “emerging” adulthood (25 years of age) [7]. Hong Kong is a Special Administrative Region (SAR) of China and its economy is characterized by free trade and low taxation. Hong Kong, which is divided into 18 districts, is one of the most densely populated places in the world (6880 people per square kilometer, as of mid-2018) [8]. In Hong Kong, approximately 93% of such individuals are considered to be “insufficiently physically active” as they fail to meet the physical activity (PA) recommendation (i.e., at least 150 min of moderate-intensity aerobic PA throughout the week, at least 75 min of vigorous-intensity aerobic PA throughout the week, or an equivalent combination of moderate- and vigorous-intensity activities) suggested by the Department of Health, Hong Kong Government [9]. Next, the issue of weight gain in youths transitioning from secondary school to their first year of undergraduate studies (“freshmen” year) is a concern, because weight gain can promote the occurrence of health risks related to being overweight [10]. Vella-Zarb and Elgar [10] asserted that while research has confirmed the vulnerability of the majority of youths gaining weight during the first year of studies in college/university, the need for further research in understanding the underlying phenomenon and finding appropriate solutions remains unmet. A meta-analysis on weight gain and youth transition from secondary school to “freshmen” year reported that approximately 60% of students gained weight in <6 months of transitioning into college [11]; moreover, the meta-analysis included databases from 1980 to 2014 and found a consistent level weight gain in students across several countries in Europe and North America, with approximately 1 in 10 students gaining at least 6.8 kg (15 lbs).

Although weight gain in itself should be taken with caution, the rate of obesity among youths has become a concern in Hong Kong, with approximately 24.15% of youths aged 15–24 years being overweight or obese [12]. Understanding youths in transition and the factors that contribute to their health is critical for preventing weight gain or obesity, maintaining healthy lifestyles, and lowering health care costs. Some researchers believe that obesity or other health-related concerns can be prevented in such populations because their lifestyle behaviors can be positively influenced while they are attending higher education institutions [13,14]. With regard to cost efficiency, health promotion interventions or other disease preventive programs targeted at this university-age population may be less costly than those targeted at other populations; this is because exercise facilities and health promotion departments or organizations usually pre-exist on campuses [13]. In addition, adolescence is a critical period during which adolescents develop attitudes and behaviors regarding health, which may be carried into adulthood [15]. Risk factors for chronic diseases, such as hypertension and inactivity, may transfer from adolescence to adulthood and limit independence during adulthood [16].

Public health scholars and practitioners have increasingly demanded studies or interventions that incorporate the socioecological perspective into their design. The social ecological model [17] emphasized that the behavior or health of an individual is not only linked to their environment but also to their social (interpersonal level) and physical environment (environmental level). Individual environment refers to personal factors, such as gender and socioeconomic status, that influence their participation in PAs. Generally, adolescents with a higher socioeconomic status are positively associated with their health [18]. Moving on, physical environment refers to both the natural and built, or man-made, environment in which one spends their time for PAs. The most supported environmental correlates of PA in adolescents, as reported by Ding et al. [19], were walkability, traffic speed/volume, access/proximity to recreation facilities, land-use mix, and residential density. Finally, social environment refers to the people, culture, and society with whom an individual interacts, such as social influencers or friends [20].

It is pertinent to consider the social ecological model of environment in conjunction with PA and youth health, as the social ecological model [17] bears on the interrelatedness of individual and environmental features such as the neighborhood setting, family, community, and the society at large [21]. Although there are health interventions based on individual characteristics, those that combine personal, community, and public policy contexts are more likely to have an effective outcome for youth health [22]. The Norwegian sample of youth health promotion in schools is an example of the interjection of individual characteristics of students, the social ecology of the school, and the outside community, an approach that provides a sustainable health intervention for youths in transition. The school environment fosters education and social values, creating a supportive environment that promotes youth learning and health [22]. Simon et al. [23] examined the effect of a school-based multilevel intervention targeting behavior and social/environmental influences on PA with the aim of preventing excessive weight gain during adolescence. This intervention was based on the dynamic interplay among PA determinants and was targeted toward not only study participants but also their family, peers, and living environment so as to promote and support PA and consequently prevent sedentary behavior. Simon et al. [23] reported that this multilevel approach prevented excessive weight gain for as long as 30 months after intervention cessation.

In Hong Kong, only few studies have examined health in individual (intrapersonal level), social (interpersonal level), and physical (environmental level) environments among youths in transition. Duan, Brehm, Chung, and Graf [24] revealed that psychosocial variables (i.e., barriers and intrinsic motivation) enhanced PA and health among university students during transition from late adolescence to adulthood in Hong Kong; however, they only examined individual and interpersonal factors. In addition, the sampling method was not randomized, lowering the generalizability of their results. More recently, Cerin et al. [25] investigated the effects of individual, social, and environmental factors as well as their interaction on obesity-related behaviors (including PA) and body mass index in adolescents aged 11–18 years in Hong Kong. In line with socioecological models of health, individual-level psychological constructs (e.g., enjoyment) and social factors (e.g., social support) could predict obesity-related behaviors. Environmental characteristics associated with neighborhood safety and/or access to public transport, destinations/facilities, and/or PA equipment were associated with increased activity levels. Nevertheless, in Hong Kong, further studies involving youths in transition (secondary school to tertiary education; 17–23 years of age) are warranted. In general, researchers have attempted to understand PA or health in the context of society, endeavoring to analyze PA within the theoretical framework of an ecological model of health, which projects an interjection of interpersonal interaction with sociocultural and environmental factors in order to improve PA levels and health behaviors [26].

In the present study, our aim was to examine the association among individual (levels of vigorous PA and frequency of using sports and recreation facilities), interpersonal (perceived social cohesion), and neighborhood environmental (availability of sports and recreation facilities) factors on the health of youths in transition in Hong Kong. Our specific hypothesis was that neighborhoods with more sports and recreation facilities as well as individuals who were more active, frequent users of sports and recreation facilities, and those with higher perceived social cohesion should be positively associated with perceived health levels. The results of this study using random sampling from youths of all Hong Kong districts may be generalized to other countries with a high population density, such as Monaco, Singapore, the Vatican City, and the Maldives. Furthermore, it will provide important information for Hong Kong urban planning and the youth commission on ways to promote youth health.

## 2. Materials and Methods

### 2.1. Participants

Herein, we targeted Hong Kong’s emerging adults, aged between 17 and 23 years in Hong Kong. The theory of emerging adulthood is a way of conceptualizing the developmental characteristics of young people between the ages of 18 and 24 years, when individuals start leaving their family of origin, with greater autonomy to assert their independence. In this study, we expanded the aforementioned conventional age range and also included 17-year-old individuals, as this is typically when emerging adults complete secondary education and transit either to the workforce or move onto tertiary education in Hong Kong.

### 2.2. Procedure

We herein used a subsample of the project “A Trans-Disciplinary Big Data Hub: Investigating the Social Mobility, Poverty and Diversity Nexus” involving adolescents. The aims of this project were to explore the social mobility outcomes of emerging adults in Hong Kong with particular attention to the concepts of childhood poverty, social capital, and ethnicity. We also looked into psychological and physical outcomes, which are believed to be tied to social mobility. In total, 508 Hong Kong local youths aged 17–23 years from 18 council districts agreed to participate and underwent a telephone survey. The survey utilized a computer-assisted telephone interviewing (CATI) system for selecting local household telephone numbers. Chiu and Jiang [27] did a study comparing residential fixed-line telephone surveys with cell phone surveys to assess the extent of the potential undercoverage issue in Hong Kong. Results found that with increasing the number of mobile phone-only households, the household telephone survey was still a better representative of the Hong Kong population as most estimates from the telephone survey sample were very similar to the population statistics. Therefore, the CATI system was chosen because it was considered reliable for the random selection of household telephone numbers and its ability to facilitate the flow of the survey questionnaire. Household telephone numbers were randomly generated using known prefixes assigned to telecommunication service providers under the Numbering Plan provided by the Office of the Communications Authority. Interviewers dialed numbers by using the CATI system to conduct interviews. If they approached an eligible person (locally born Chinese, aged 17–23 years), they would continue to conduct the interview; however, if the person who answered the call was not eligible, the interviewer would invite an eligible household member to answer survey questions. The telephone number was marked as “not qualified” if no household member was aged 17–23 years. For household members whose age was younger than 18 years, their parental consent was sought before he or she responded to the questions.

In a 1-hr interview meeting, all interviewers were made to understand questionnaire guidelines before the actual telephone interview was conducted. A protocol was provided to them that guided them in a stepwise manner on how to complete the interview. All participants were provided with information about the confidentiality of the study and were informed of their right to terminate the project at any time. Prior to administering the questionnaire, the institutional review board of the university provided us with permission to conduct this study with human subjects (REC/18-19/0039).

### 2.3. Measures

#### 2.3.1. Individual Level (Individual Environment)

Frequency of using sports and recreation facilities: this parameter was measured using the following question: “In a typical month, how often do you use facilities provided by the Leisure and Cultural Services Department of Hong Kong Government (e.g., basketball court, badminton court, library, and parks) in your neighborhood?”. All respondents were asked to answer this question on a scale of 1 (never) to 6 (almost daily).

Vigorous PA and perceived health: with regard to vigorous PA, respondents reported the number of days they had engaged in ≥20 min of vigorous cardiovascular activities that would lead to a higher heart rate (e.g., jogging). This question was developed by the American College Health Association (ACHA, 2012). Its validity has been established based on comparisons with other national databases such as the College Health Risk Behavior Survey, whereas its reliability has also been supported (Cronbach’s alpha = 0.86) (ACHA, 2012). Next, the outcome variable “perceived health” was measured by how respondents described their health using the 5-point Likert scale, ranging from 1 (excellent) to 5 (poor).

#### 2.3.2. Interpersonal Level (Social Environment)

Perceived social cohesion: this parameter was assessed using the 7-item Social Cohesion and Trust Scale [28]. Participants were provided with five statements (e.g., “this is close-knit neighborhood”) and asked to respond using the 5-point Likert scale, ranging from 1 (strongly disagree) to 5 (strongly agree). The validity was supported depending on the significant association with another related measure—informal social control (r = 0.80).

#### 2.3.3. Physical Environment (Neighborhood Environmental Level)

Availability of sports and recreation facilities: the Geographic Information Systems (GIS) software package ArcGIS v10.7 (Environmental Systems Research Institute) was utilized for the measurement of the objective physical environment. Before calculating the GIS built environment variables, all participants were address geocoded, based on the recommended constituency areas boundary descriptions. This geocoding process created a spatial coordinate for each address, allowing ArcGIS to determine our objective physical environment variables. The studied variables included the following: (i) number of other recreational venues (e.g., parks), (ii) number of sports centers, and (iii) number of swimming pools. Buffers are boundaries created at interval distances around the home of each participant. In this study, a constituency was used to delineate the neighborhood of each subject.

### 2.4. Sociodemographic Characteristics

The characteristics of respondents in this study included age, gender, education level (i.e., primary education or below, secondary school, tertiary education or above), income (i.e., family household income), and length of residence…etc.

Before interviews were conducted, all questions were developed through our qualitative pilot study that was conducted in August 2017. Two interviewees were recruited using the snowball purposive sampling method. The survey procedures, clarity of research questions, and collected data were reviewed by researchers. There were no major changes in the question guides and questionnaire after the pilot study.

### 2.5. Data Analysis

Data management and analyses were performed using the maximum likelihood estimation with Mplus v8.3 (Muthén & Muthén, Los Angeles, CA) and SPSS v21.0, with the *p* value set to 0.05. Structural equation modeling (SEM) was used to examine the association among individual (vigorous PA and frequency of using sports and recreation facilities), interpersonal (perceived social cohesion), and neighborhood environmental (availability of sports and recreation facilities) factors on the health of youths in transition in Hong Kong. SEM was used because it enables measurement errors of observed variables to be analyzed as an integral part of the model. Multiple fit indices suggested by Kline [29] were used to assess the model fit. Both absolute and relative fit indices were involved in data analyses. The absolute fit indices included the chi-square value, root-mean-square error of approximation (RMSEA), and the standardized root mean residual (SRMR). SRMR and RMSEA values < 0.08 generally indicate acceptable fits and relative chi-square values ranging from 2.0 to 5.0 are considered desirable [30]. Comparative fit index (CFI) values > 0.90 indicate a good fit to the data. In addition, standardized factor loading, standardized error, squared multiple correlation (R2), and *t* values were inspected [31].

Of 508,206 participants were excluded from data analyses due to incomplete information for address geocoding. The eventual sample size met the recommended sampling requirement to maintain an adequate sample size-to-parameter ratio for structural modeling [32]. Both individual and neighborhood level variables were analyzed within a multilevel path analysis framework. Mplus v8.3 (Muthén & Muthén, Los Angeles, CA) was used for the analysis of this multilevel model. All model tests were based on the correlation matrix and used maximum likelihood estimation.

## 3. Results

Participants’ sociodemographic characteristics are summarized in Table 1, and descriptive statistics and interrelations for all studied variables are presented in Table 2. In total, 211,295 telephone numbers were dialed, in which 175,974 numbers were valid telephone numbers but inaccessible due to unanswered calls or busy line; 31,264 numbers had no eligible person (locally born Chinese, aged 17–23 years) in the household for the interview. Among the 4057 eligible people we reached, 508 provided consent and completed the interview, resulting in a response rate of 12.5% (508/(3549 unsuccessful cases + 508 successful cases). This low response rate might be due to the length of survey (about 1 hr) and participants’ inclusive criteria (17–23 years old). Therefore, our response rate was lower than that (about 50%) of other Hong Kong telephone surveys targeting the general population (i.e., 18 years or older) [27]. Still, our study’s response rate was closer to that of another study [33] using telephone survey in Hong Kong (17%) and within the range of response rates for telephone polling in the US [34].

Of 508 Hong Kong local youths aged 17–23 years from 18 council districts that participated our study, 302 individuals with complete data pertaining to address geocoding were selected for further analyses. Of among 302 youths (147 males and 155 females), only 24.8% had attained tertiary education or above and 63.9% of them were full-time students. Their average age was 19.72 (SD = 2.04) years. Approximately 52.3% of participants were living in subsidized housing and 82.5% of them had lived there since birth. About 36.42 of them had a monthly household income, ranging USD 2564–USD 7692. More importantly, 50% (56.3%) of participants used sports and recreation facilities, such as a basketball court, badminton court, library, or park, only once per month or even less. Among this group of participants, 39.1% never used these facilities at all in a month. Furthermore, as per the PA recommendation suggested by the WHO [35], only 15.10% and 27.49% participants engaged in moderate (i.e., at least 150 min of moderate-intensity aerobic PA throughout the week) and vigorous (i.e., 75 min of vigorous-intensity aerobic PA throughout the week) PA. On average, participants engaged in moderate and vigorous PA for 2.23 (SD = 1.88) and 1.16 (SD = 1.78) days, respectively, in a week.

**Figure 1:**
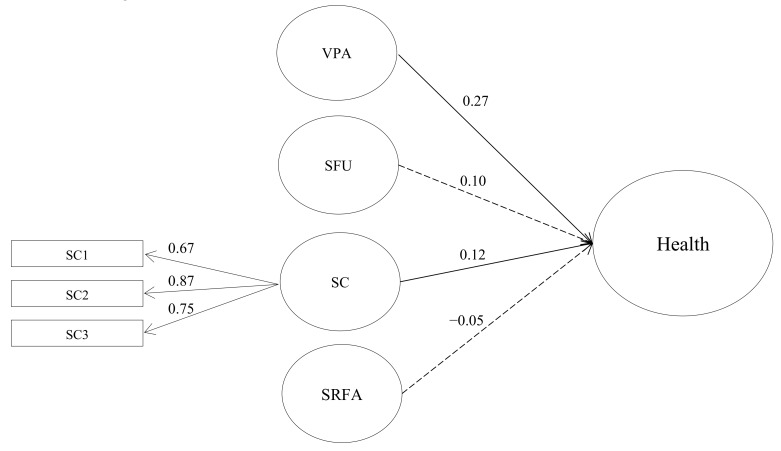
Final Structural Model. Note: Doted lines represent insignificant parameter while solid represent significant parameter. Health = perceived health; SFU = sport facilities usage; SC = perceived social cohesion; SRFA = sports and recreation facilities availability.

Except for perceived social cohesion (five items) that was tested as a latent variable, other variables (levels of vigorous PA, frequency of using sports and recreation facilities, and availability of sports and recreation facilities) were tested as observed variables. SEM results indicated that the proposed model sufficiently fitted the data (χ^2^ (24) = 32.23, *p* < 0.12; CFI = 0.977; SRMR = 0.051; RMSEA = 0.034 (90% CI = 0.000–0.061)). However, two items of perceived social cohesion, namely “people in this neighborhood do not share the same value (SC4)” and “people in this neighborhood generally do not get along with each other (SC5)” were sequentially removed one by one due to low standardized factor loadings (<0.3). A structural model was reinserted into the data analysis, and the modified model fitted the data well, as indicated by fit indices (χ^2^ (11) = 15.29, *p* < 0.17; CFI = 0.987; SRMR = 0.054; RMSEA = 0.036 (90% CI = 0.000–0.075)). Results showed that both vigorous PA (*β* = 0.27, *p* = 0.0005) and perceived social cohesion (*β* = 0.12, *p* = 0.048) were significantly related to perceived health at an individual level. Furthermore, PA and perceived social cohesion were responsible for an 11% variation in perceived health. Physical environment (i.e., number of available sports and recreation facilities, *β* = 0.098, *p* = 0.09) and frequency of using sports and recreation facilities (*β* = −0.052, *p* = 0.34) were not significantly related to perceived health. The standardized results for each of the main structural parameters defined in the model are summarized in Figure 1.

## 4. Discussion

Herein, we examined the effect of predictors (individual factors (levels of vigorous PA and frequency of using sports and recreation facilities), interpersonal factor (perceived social cohesion), and neighborhood environmental factors (availability of sports and recreation facilities)) on the health of youths in transition in Hong Kong. We found that only vigorous PA and perceived social cohesion were positively associated with health.

### 4.1. Vigorous PA and Health

The obtained result was in line with our expectations that PA promotes youth health. In a systematic review [36] and study [37], authors investigated the short- and long-term health effects of PA in adolescents and found that PA was, indeed, beneficial as it enhanced cardiorespiratory fitness, lowered body fat, improved peak bone mass, increased self-esteem, lowered stress levels, and reduced anxiety sensitivity. Specifically, in this study, only the effect of vigorous PA was measured, and our findings concur with the existing evidence that vigorous PA is more strongly associated with health outcomes in young people compared with PA of other intensities such as light- or moderate-intensity PA [38,39]. Schilter and Dalleck [40] also found that vigorous PA enhanced health status because it lowered the resting heart rate, increased high-density lipoprotein levels, and reduced fasting blood glucose levels among university students. In fact, almost no studies have yet elucidated mechanisms underlying the positive benefits of vigorous PA on youth health; the suggested explanation may include changes in fitness levels and body shape, in addition to improved self-efficacy and physical self-concept [38]. Other possible mechanisms in youths could be related to an increased opportunity to participate in interuniversity or interschool competitions in Hong Kong as well as excitement or emotional arousal from vigorous PA.

### 4.2. Social Cohesion and Health

Corresponding to the findings of previous studies [41,42], our SEM results revealed that social cohesion could predict perceived health levels. Accordingly, social cohesion seems to be gaining popularity in the public health literature. Bjornstrom, Ralston, and Kuhl [43] explained that individuals with perceived higher levels of social cohesion may display feelings of solidarity, safety, or prosocial consciousness; this in turn reduces stress and has health benefits. Still, Yu et al. [44] highlighted that mechanisms responsible for the association between social cohesion and health are inconclusive. Other possible pathways of linkage between social cohesion and health include social support (i.e., facilitating access to health information and services) [45] and offsetting the negative effects of stressors on mental health [46]. Additional studies should aim to investigate mechanisms underlying such an association.

### 4.3. Effects of Availability and Frequency of Using Sports and Recreation Facilities on Health

In this study, both frequency of using and availability of sports and recreation facilities did not predict youth health. Some studies have evaluated the association between the availability of sports and recreation facilities and PA and health among youths [47]. For instance, Black, Shield, Propper, and Johnson [48] examined the impact of attending a school with inadequate sports facilities on the health of adults later in their life; they found that attending such a school had no identifiable effects on physical health. However, other studies have reported that more favorable environments, such as objective availability of parks [49] and recreational facilities [50,51], can enhance participation in PA and consequently promote health.

With regard to the frequency of using sports facilities, a direct relationship has been reported to exist between people who frequently exercise and use sports facilities and health-related quality of life [52]. However, this was not the case in this study. One of the possible explanations for this discrepancy could be that we only measured the frequency of using public sports and recreation facilities. It is possible that our study participants engaged in sports or PA in sports facilities that were not provided by the Leisure and Cultural Services Department of Hong Kong Government, such as private or school sports and recreation facilities. Moreover, the frequency of using sports and recreation facilities does not accurately reflect key factors such as the duration and intensity of PA. Hence, people who may frequently use such facilities could often perceive their health levels to be low if the duration and intensity of PA were less.

### 4.4. Practical Implications

Our results provide important information for urban planning and the youth commission on ways to promote youth health. We found a positive association between vigorous PA and youth health. While (1) frequency of using and availability of public sport and recreation facilities were not associated with youth health and (2) vigorous PA predicted youth health, in future, researchers or practitioners should examine, for example, factors that facilitate or obstruct involvement in vigorous PA in youths and locations in which youths perform vigorous PA.

Secondary schools or universities provide a perfect outlet or venue for students to acquire technical knowledge on muscle training, bodybuilding, and competitive sports training. Education institutions may consider developing more vigorous PA interventions or programs, such as developing credit or non-credit bearing courses and launching more intramural, interuniversity, or secondary school competitions. The content of the course could include details pertaining to PA recommendations and guidelines as well as behavior change strategies (e.g., goal setting, positive self-talk, and logging strategies). Specifically, the association that exists between university recreation centers and university health services provides an opportunity for campus partnerships focused on enhancing the knowledge of students on vigorous PA and increasing their interest to participate in such activities. Ideally, academic departments such as kinesiology and physical education may join such campus partnerships and provide them with academic support. King et al. [53] examined vigorous PA behavior among university students using the health belief model [54], and they found that perceived benefits and cues to exercise were associated with more vigorous PA involvement, while perceived barriers were associated with less vigorous PA involvement. The top three perceived barriers were school workload, lack of motivation, and jobs, whereas the top three perceived cues were looking physically fit, looking at oneself in the mirror, and having an exercise partner. Future vigorous PA programs should therefore consider ways to tackle these barriers and incorporate such cues. In urban planning, the government may provide more physical outlets for youths to do vigorous physical activity. Street fitness, or street workout, is popular these days. Street workout is a type of vigorous physical activity or fitness training, combining gymnastics and parkour. To the youth, street workout is costless because it can be done nearly anywhere in a park or open area. The government may, therefore, investigate its required facilities and accommodate them in neighborhoods. Of course, the government may organize more vigorous PA programs for those youths outside education settings.

Our study also revealed that social cohesion predicted youth health. To increase social cohesion, the government should allocate more resources to develop community programs, which tend to help people learn more about their neighborhood, engage with their neighborhood, as well as identify and deal with possible issues that may undermine social cohesion (e.g., class discrimination) in their neighborhood. Moreover, to develop these programs, the government should create a strategy to first make an initial contact and then build partnerships with businesses or community groups. For example, the government may opt to work in partnership with some education institutions or sports associations to keep the youth active and promote the use of sports for building meaningful and more cohesive, trustful relationships among people in the neighborhood.

## 5. Conclusions

We examined the effect of predictors (individual factors (levels of vigorous PA and frequency of using sports and recreation facilities), interpersonal factor (perceived social cohesion), and neighborhood environmental factors (availability of sports and recreation facilities)) on the health of youths in transition in Hong Kong. We found that only vigorous PA and perceived social cohesion were positively associated with health.

To the best of our knowledge, this is one of the few studies to examine the health of youths in transition in Hong Kong using multiple levels of the social ecological model. As few related studies have been conducted in Hong Kong, we believe that our study results can facilitate the generalizability of existing research findings from Western countries. Herein, random sampling was used to select a sample of 508 individuals aged 17–23 years old from all council districts (18 council districts) in Hong Kong, which is the strength of this study regarding our finding’s generalizability. Nonetheless, this study has some limitations. First, we did not address the causality direction in this cross-sectional study. Second, the validity of the PA question may be affected by the knowledge of participants on vigorous PA. Third, questions asked in the survey were limited, hampering our ability to measure or apply some constructs. For instance, there was only one item measuring perceived health. Asking about different domains of health, such as social health or psychological health, may provide further insights into our studied discipline. Forth, the low response rate (12.5%) may lower the representativeness of the sample. Further studies employing multiple levels of the social ecological model to explore the health of youths in transition are warranted; moreover, its highly advisable to include other health variables such as obesity, quality of life, and mental health.

## Figures and Tables

**Table 1:** Sociodemographic Characteristics of Participant (n = 302).

Characteristic	N or Mean
Sex	
Female	155
Male	147
Length of Residence in Hong Kong	
Since birth	249
Others	53
Mean Age (years)	19.72 (SD = 2.04)
Education level	
Primary School Level	2
Secondary School Level	225
Tertiary education and Above	75
Students	
Full-time Students	193
Part-time Students	5
Non-students	104
Types of housing	
Subsidized housing	158
Non-subsidized housing	138
Household income (HKD)	
9999 or less	5
10,000–19,999	17
20,000–29,999	39
30,000–39,999	30
40,000–59,999	41
60,000–99,999	17
100,000 or above	10
Refuse to respond	143

Note. N = number of participants; SD = standard deviation; Exchange rate USD 1 = HKD 7.7670.

**Table 2:** Descriptive Statistics and Interrelations for All Variables Studied.

	M (SD)	Health	SFU	SC1	SC2	SC3	SC4	SC5	VPA
Health	3.05 (1.038)								
SFU	2.43 (1.478)	0.176							
SC1	2.99 (0.973)	0.080	0.171						
SC2	3.42 (0.923)	0.119	0.143	0.582					
SC3	3.39 (0.833)	0.128	0.118	0.501	0.646				
SC4	2.34 (0.769)	−0.031	0.082	−0.060	−0.080	−0.021			
SC5	3.67 (0.836)	0.043	0.007	0.119	0.254	0.272	0.093		
VPA	1.59 (1.784)	0.294	0.239	0.039	0.027	0.016	0.019	−0.087	
SRFA	0.55 (0.842)	−0.060	0.046	−0.044	0.062	0.045	−0.056	0.021	−0.067

Note. M (SD) = Mean (Standard deviation); Health = perceived health; SFU = sport facilities usage; SC1 = close knit neighborhood; SC2 = help from neighbors; SC3 = trust in neighborhood; SC4 = people not having same value in neighborhood; SC5 = people not getting along in neighborhood; VPA = vigorous physical activity; SRFA = sports and recreation facilities availability.
